# The True and the False

**Published:** 1889-12

**Authors:** 


THE TRUE AND THE FALSE.—When the Savior of the World was arraigned before Pilate and falsely charged with blasphemy and other crimes, the Roman Governor put to him that memorable question, "What is Truth?" Happy it would have been for Pilate if he had given his decision at his tribunal on the side of truth, instead of cowardly yielding his conviction to the voice of popular clamor.

But policy, that baneful principle to the righteousness of private and public questions, prevailed, and under its pressure he committed the fearful deed of giving the sinless Christ into the hands of the blood-thirsty mob.

As in the days of the Roman Governor, so in all ages, falsehood sometimes has successfully dominated truth for a season, but in every instance it suffers ignominious defeat in the end, while truth recovers and receives triumphant vindication.

The writer has often thought it strange that men of intelligence did not know and apparently did not see that this is the inevitable result which they must sooner or later reach when they cultivate principles, or use methods and means which are antagonistic to truth and right-doing. Because they meet with an insecure and questionable success for a time they seem to lose sight of the end, and to be unconscious of the fact apparent to common sense, that this end must at last prove to be their undoing in one way or another. They seem to apprehend but one thing, and to guard against but one result, and that is being "found out," but not because they fear so much the exposure of wrongdoing as the loss of temporal gain that the revelation will bring to them.

We hear of different grades of falsehood, but I do not think there are any such offences as fibbing, "white lies," etc. A thing is either true or false; it cannot be both, when the two are diametrically opposite in their nature.

I once heard the great and good Bishop, George H. Pierce, of Georgia say, in one of his grandly eloquent sermons, that a man could be guilty of a lie without opening his lips to give it utterance, that an insinuation, or the intention and the act to deceive and mislead was falsehood, as surely as if the person had told it in so many words.

It is the character of falsehood to be servile, treacherous and underhanded, loving to work in the dark, but truth in its purity and beauty, seeks the effulgent light of day, and standing out fearlessly before the eyes of individuals and the world, it challenges the closest scrutiny to which one may desire to subject its stainless character. Unlike falsehood, it does not evolve its principles in the shadow of some hidden corner, for it has nothing to conceal, but is glad and anxious to make known its every method and demonstration. Truth, also, has a sublimity about it to which even falsehood bows in reverence, and at the same time, it is so simple and direct as to be evident to the mental perception of a child, and it never needs a glittering array of chosen words to carry its conviction home to the heart of him to whom it is directed. Falsehood may leave itself a wide margin, but truth has more, and no sane man can take any question and say "I did not know, or had no means of knowing which was right or wrong, true or false."

Of all professions we should have true men in that of medicine, men who will stand firmly by every conviction of right and justice, true to their friends, appreciative and grateful for favors granted, true to the honor and interest of the profession. The man who is destitute of gratitude is wanting in one of the crowning virtues of life, and is unworthy of the esteem and confidence of all good men.

True men in the profession will command honor and respect from the intelligent and worthy; they will seek to give an impetus to its progressive achievements, and thus enlarge its capacity for good to humanity, and at the same time elevate their own character to that noble standard to which it is their duty and to which all the best men of the fraternity are desirous that the members of the profession should attain.

